# When the hospital becomes the battlefield: patients as stakeholders in mass casualty incidents

**DOI:** 10.1186/s13584-025-00728-x

**Published:** 2025-11-10

**Authors:** Galia Karp Kahana, Shlomi Codish

**Affiliations:** 1 Soroka Medical Center Management, Hospital Administration, Soroka Medical Center, Beer-Sheva, Israel; 2 Department of Health Policy and Management, Ben-Gurion University of the Negev, Beer-Sheva, Israel

**Keywords:** Patient participation, Emergency preparedness, Mass casualty incidents, Hospital disaster resilience

## Abstract

**Abstract:**

Hospitals are uniquely positioned during disasters, functioning as first-line responders and at risk themselves. While system preparedness and staff resilience have been widely studied, the experience and preparedness of patients admitted to hospitals during mass casualty incidents (MCIs) have received little attention.

This commentary was prompted by the recent study of Wolff et al., which assessed inpatients’ knowledge of how to respond during an MCI and the barriers they perceived in such situations. Their work highlights gaps in patient awareness and preparedness, raising the question of whether and how inpatients should be involved in disaster response. Drawing on the experience of Soroka Medical Center during the June 2025 Israel–Iran conflict, we reflect on these findings in the context of a real-world missile strike on a major tertiary medical center.

On June 19, 2025, Soroka was directly struck by a ballistic missile that destroyed a surgical inpatient building, rendering 562 beds unusable and necessitating mass evacuation and transfers. The event revealed the complexity of patient reactions—from acute fear and uncertainty to pragmatic acceptance of crowding in compromised conditions. Hospital leadership rapidly instituted patient education on air-raid protocols, relocated vulnerable patients, and implemented staff refresher training. Despite these efforts, challenges in communication, rumor control, and logistics significantly affected inpatients’ sense of safety and continuity of care.

**Conclusions:**

Wolff et al.’s study and Soroka’s lived experience converge on a critical insight: patients cannot be passive bystanders in disasters. Their perceptions, behaviors, and resilience have a direct impact on hospital functionality. Future research should define the scope of patient roles in preparedness, assess the psychological impact of preparedness training, and inform scalable policies for integrating patients into hospital resilience frameworks. Incorporating patient preparedness into disaster planning is crucial, especially during times of heightened alert and in regions prone to conflict or natural disasters.

The 12-day war between Israel and Iran, known in Israel as Operation Rising Lion, began on June 13, 2025. In the ensuing days, Iran fired over 500 ballistic missiles at Israel. Several of these struck various targets, including Soroka Medical Center in Beer-Sheva, Israel, which was directly hit on June 19, 2025, the seventh day of the war. The missile destroyed a surgical inpatient building, damaging surrounding buildings, rendering 562 patient beds (about half of Soroka’s capacity) unusable, and necessitating the evacuation of 287 patients from these buildings. This single event highlighted, in the starkest possible terms, the complex reality hospitals face as both first-line responders in mass casualty incidents (MCIs) and potential targets.

Hospitals, their staffs, and their patients face considerable risks in situations that include both human-caused disasters, such as war, and natural disasters. Threats include internal disruptions, such as strikes, and external threats, such as direct structural damage from bombardment or seismic events, disruption of critical lifelines (such as electricity, water, oxygen, and information systems), and the sudden loss of access routes for staff, supplies, and emergency services [1]. Unlike many institutions, hospitals cannot suspend operations during crises. Patients on life support, in intensive care, or receiving dialysis require uninterrupted care, and evacuation is often infeasible [2]. In conflict settings, hospitals may also become symbolic targets, with attacks aimed at undermining public morale and crippling a community’s ability to respond to mass casualties. In the context of natural disasters, vulnerabilities stem from aging infrastructure, the location of critical systems in basements prone to flooding, and non-retrofitted buildings in seismic zones [3]. Previous direct hits on medical centers in Israel were considerably smaller in scale, both in payload and in extent of damage. While these risks to hospitals are widely addressed in the literature, far less attention has been devoted to the preparedness, responses, and needs of inpatient populations when a hospital becomes the epicenter of a disaster [4].

The reactions of patients already admitted to a hospital during a mass casualty incident are complex and often profound. The sudden influx of casualties can create an atmosphere of chaos, fear, and uncertainty, intensifying distress among exposed individuals [5]. Operationally, situations of heightened risk can necessitate the relocation of inpatients to protected areas and the suspension of routine procedures, amplifying their sense of vulnerability [6]. These dynamics suggest the need to integrate rapid mental health identification, triage, and support into hospital disaster response plans [7]. In addition, patient perceptions are significantly shaped by rumor, trust, and communication—a pattern observed in disasters worldwide, from earthquakes in Turkey to attacks in Ukraine. Poor communication tends to increase fear, whereas clear, timely, and authoritative communication can help mitigate panic [8].

Previous research has primarily focused on system-level responses to MCIs and their effects on healthcare delivery and staff. Research on the effects of MCIs on hospitalized patients—whether the event is external or occurs within the hospital—is sparse [9].

In a recent article in this journal, Wolff et al. addressed this important aspect of patients’ responses to mass casualty incidents [10]. Conducted outside of an immediate threat scenario, the study assessed patients’ knowledge of how to respond during an MCI and the barriers they perceived at the hospital where they were inpatients. Their results reveal gaps in the knowledge of the inpatient population and their preparedness for such events. Since patient turnover is high, it may seem infeasible to routinely train or educate patients on MCIs, but doing so appears crucial in times of elevated tensions or increased risk for disasters. This situation raises ethical and practical questions. On the one hand, patients must not be overlooked in emergency preparedness efforts. On the other hand, we must ask ourselves whether inpatients should be expected to acquire disaster-response skills. Where is the line between empowering patients and burdening them with responsibilities that should remain within the purview of healthcare professionals?

At Soroka, from the onset of the 12-day war, we evacuated departments located in unprotected areas and relocated vulnerable patients with mobility limitations—even from relatively safe locations—to better-protected areas. The relocations were carried out iteratively as spaces became available, and as the threat of Iranian ballistic missile strikes became increasingly evident. The most dramatic of these was the evacuation of our urology and ENT departments just 16 h before the missile slammed directly into their original locations.

Medical teams were instructed on the areas offering maximal protection in each ward. Subsequently, patients received guidance on how to respond to air-raid alerts. In cases where patients were unable to care for themselves or understand these instructions, their caregivers were instructed. Staff were directed to care for unattended patients with mobility or cognitive limitations ahead of other patients during air-raid alerts. To some extent, these activities mitigated the knowledge and preparedness deficiencies described by Wolff et al. Patient crowding and substandard admission areas posed challenges to the quality of care, but patients accepted this situation, considering the ongoing war.

We provided staff with frequent updates about security risks. On Tuesday, June 17, 2025, we decided to refresh our preparedness for earthquake protocols to enhance readiness for the possibility of a missile strike without directly referring to missile fire to prevent panic. This training highlighted the importance of the clinical leader in each department immediately after a disaster occurs until the emergency team and management issue clear instructions. This guidance was not given to patients, and it is unclear whether it would have been beneficial or potentially confusing. Once again, a central unanswered question arises: How much information is enough to empower patients without overwhelming them or undermining staff authority amid chaos?

Following the missile strike, patients and staff members were affected in several ways, as described below:

*Immediate physical effects* – The blast effect of the 750 kg warhead was felt across the entire medical center. There was a significant amount of debris and damage. In addition, unfounded early media reports of hazardous materials and an alleged radioactive leak caused concern among patients and staff until we were able to clarify the situation officially.

*Rapid discharge and transfer* – With 562 beds rendered unusable, patients were either discharged or transferred to hospitals across the country. Evacuation on stretchers via intact stairways was necessary, since all elevators were dysfunctional due to the blast. There were 861 patients at Soroka at 7:13 am on June 19. Of these, 287 were hospitalized in severely damaged buildings. Following the missile strike, 157 were discharged to the community, and 96 were urgently transferred to continue care at other medical centers across the country. Thirty-four patients remained at Soroka and were transferred to other buildings.

*Numerous logistics challenges for remaining patients* – For example, the destruction of our central kitchen necessitated bringing in food for both staff and the remaining patients. Similarly, the loss of the central pharmacy required us to rely on medications stored in remaining units and supplies provided by other medical centers.

Another logistics challenge was the transportation route to our remaining operating rooms when the elevators were not functional. We used ramps built in the 1950s from the sub-basement (level − 2) to the first-floor operating rooms—designed precisely for such a scenario but used for the first time during the war out of necessity.

*Mental health support for patients* – In accordance with our protocol, we opened a stress treatment site in our Emergency Department for those affected by the missile strike. However, the urgent need to evacuate patients—either to the community or to other medical centers—limited our ability to provide ongoing mental health support.

*Emotional and morale challenges for medical teams* – For our over 5,700 workers, Soroka Medical Center is much more than a workplace. It is not surprising that, contrary to the natural human instinct to flee from a disaster area, workers rushed from home to assist in rescue and evacuation. Shortly after the missile strike, the extensive destruction and the loss of several wards caused emotional distress for a significant portion of our team, with long-lasting effects for some. Our resilience team and Mental Health Department are overseeing our recovery. This process is ongoing and continues alongside the physical renewal and rebuilding.

The study by Wolff et al. highlights the importance of including patients in real-time emergency preparedness during emergencies and times of heightened alert. Future research is needed to determine the optimal level of patient inclusion, the appropriate areas for patient guidance, and whether patients should assume a formal role in such emergencies.

We suggest that future studies explore three critical dimensions: (a) defining the scope of patient roles in disaster response, (b) evaluating the psychological effects of disaster-preparedness education on vulnerable patients, and (c) establishing scalable policies that integrate patient preparedness into national hospital resilience frameworks.

This commentary adds to the growing recognition that patients are not passive bystanders in disasters. Their preparedness, perceptions, and resilience must become an integral component of hospital emergency planning.

## Data Availability

No datasets were generated or analysed during the current study.

## Abbreviations

MCI: Mass casualty incident
